# Identification of B and T Cell Epitopes to Design an Epitope-Based Peptide Vaccine against the Cell Surface Binding Protein of Monkeypox Virus: An Immunoinformatics Study

**DOI:** 10.1155/2023/2274415

**Published:** 2023-02-22

**Authors:** Lincon Mazumder, Md. Rakibul Hasan, Kanij Fatema, Shamima Begum, Abul Kalam Azad, Mohammad Ariful Islam

**Affiliations:** Department of Microbiology, Jagannath University, Dhaka 1100, Bangladesh

## Abstract

**Background:**

Although the monkeypox virus-associated illness was previously confined to Africa, recently, it has started to spread across the globe and become a significant threat to human lives. Hence, this study was designed to identify the B and T cell epitopes and develop an epitope-based peptide vaccine against this virus's cell surface binding protein through an *in silico* approach to combat monkeypox-associated diseases.

**Results:**

The analysis revealed that the cell surface binding protein of the monkeypox virus contains 30 B cell and 19 T cell epitopes within the given parameter. Among the T cell epitopes, epitope “ILFLMSQRY” was found to be one of the most potential peptide vaccine candidates. The docking analysis revealed an excellent binding affinity of this epitope with the human receptor HLA-B^∗^15:01 with a very low binding energy (-7.5 kcal/mol).

**Conclusion:**

The outcome of this research will aid the development of a T cell epitope-based peptide vaccine, and the discovered B and T cell epitopes will facilitate the creation of other epitope and multi-epitope-based vaccines in the future. This research will also serve as a basis for further *in vitro* and *in vivo* analysis to develop a vaccine that is effective against the monkeypox virus.

## 1. Introduction

While millions of deaths were reported worldwide due to the SARS-CoV-2 pandemic [1], a new zoonotic disease, monkeypox (mPox), caused by the monkeypox virus (MPV), has become a severe public health concern. The monkeypox virus, closely related to the variola virus that causes smallpox, was initially identified in monkeys in 1958 [2] and in humans in a forested area of central Africa in 1970 [3, 4]. While mPox is endemic in parts of west and central Africa, its recent occurrence in a number of nonendemic locations outside of Africa has caused public health officials to express grave concerns [5]. Since smallpox was eradicated due to widespread vaccination, there have been very few natural outbreaks of the Orthopoxvirus until recently [2]. However, after the confirmed outbreaks in over 70 countries where the virus has not been reported before, the World Health Organization on July 23 issued its highest level alert, designating mPox as a public health emergency of international concern.

The MPV belongs to the genus Orthopoxvirus and in the family Poxviridae [2]. Currently, two variants of mPox in human are widely known. The Central African or Congo Basin variant (clade I) of mPox, which has a fatality rate of up to 10%, is the more severe of the two forms of mPox that affect humans [6]. In contrast, the West African variant (clade II), responsible for the current outbreak, causes a less severe disease with a lower mortality rate of 3% or less [4, 7]. The mPox can be transmitted among the community through a number of methods. The nosocomial and home routes of mPox transmission between people are extensively known, while sexual contact has also been proposed as a potential means of transmission [8, 9]. The disease can spread from one person to another if they are in close proximity to an infected individual's skin sores, contaminated bedding, or big respiratory droplets [10]. Aside from the complications like pneumonitis, encephalitis, sight-threatening keratitis, and secondary bacterial infections, the typical clinical syndrome of mPox includes fever, rash, lymphadenopathy, and other symptoms [8]. The prominent-risk individuals of mPox sickness are immunocompromised individuals, younger people, elderly people, pregnant women, and people with diabetes and HIV/AIDS [5, 11].

Previously, smallpox vaccine Dryvax was most commonly used to treat both smallpox and mPox [12, 13]. Since late 2019, the COVID-19 pandemic has had a tremendous impact on the entire world, unequivocally highlighting the necessity of prophylactic measures in the early phases for the emerging viruses like MPV. Developing a vaccine capable of eliciting a sufficient immune response, effectively combating virus colonization and proliferation in the host, and eradicating the virus that has been introduced into the host can be a practical preventative approach to combat mPox virus-associated illness in the early stages. The MPV possesses a number of virulence factors which may help the virus to propagate within the host cell and circumvent the immune defenses. Shantier et al. reported that the cell surface binding protein of mPox plays a role in the pathogenesis of the virus [13]. The MPV virion membranes contain the cell surface binding protein E8L, which plays a crucial role in the attachment to the host cells [14]. This MPV protein attaches to chondroitin sulfate on the cell surface to enable virion attachment to the target cell [15]. Yousaf et al. also reported that it is produced in the outer membrane of the virus and helps in virion attachment to the target cell by binding to chondroitin sulfate on the cell surface [16]. As a result, the cell surface binding protein may be a promising vaccine target in order to create a unique and safe vaccination capable of triggering the desired immunological response. In order to understand disease etiology, monitor the immune system, develop a diagnostic assay, and design a vaccine, it is crucial to recognize epitopes in antigens [17]. During the recent outbreak of SARS-CoV-2, a number of vaccine candidates were proposed based on the identification of B cell and T cell epitopes. In an experiment by Singh et al., for example, they implemented immunoinformatics to develop a vaccine against the spike surface glycoprotein of SARS-CoV-2 using epitope-based peptide vaccines with high confidence [18]. Similar to the ebola virus, which is an antisense-strand RNA lethal virus that caused an epidemic, Dash et al. proposed an epitope-based vaccination against the virus's glycoprotein [19]. In addition, a potential vaccine against the dengue virus, which poses a global health threat, was developed by Fadaka et al. using suitable adjuvants and linkers in addition to the selected epitopes [20].

This study is aimed at identifying the B and T cell epitopes on the cell surface binding protein and at designing an epitope-based peptide vaccine against the MPV by utilizing several computational databases and bioinformatics tools and software. Besides, this study is aimed at investigating the evolutionary relationship of this virulence protein with other similar types of proteins, as well as predict the protein's three-dimensional structure and its refinement and quality assessment. Molecular docking analysis and molecular dynamic simulation were further employed in this study to investigate the interactions between the vaccine and host cell receptor as well as its stability. The outcome of this research will therefore enhance our knowledge to investigate potential therapeutics for emerging and reemerging pathogens and will serve as a basis for further *in silico* studies and benefit to the researchers.

## 2. Materials and Methods

### 2.1. Study Protocol Employed in This Study

**Figure 1:**
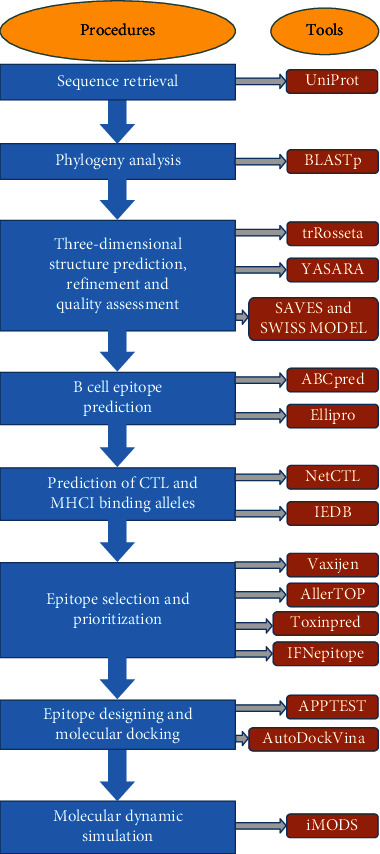
Summarized protocol used to design a single epitope-based peptide vaccine against mPox virus.

The current study employed computational databases and bioinformatics tools (depicted in Supplementary Table 1) to design an epitope-based peptide vaccine against the MPV. The workflow in Figure 1 represents the entire methodology for the final construction of the vaccine.

### 2.2. Retrieval of the Sequence and Phylogeny Analysis

The mPox viral protein sequence was retrieved as a FASTA file from the UniProt database (https://www.uniprot.org/) [21] by searching with the keyword “monkeypox cell surface binding protein.” Following that, we used our target sequence as the query sequence to analyze the BLASTp program [22] for evaluating phylogenetic information from the NCBI database (https://www.ncbi.nlm.nih.gov). Finally, in order to better understand the comparative evolution of the organisms, a phylogenetic tree was constructed and visualized using the Microreact database (https://microreact.org/) [23].

### 2.3. Three-Dimensional Structure Prediction, Energy Minimization, and Quality Assessment

A web-based program called trRosetta, which predicts protein structures fast and accurately, was used to construct the three-dimensional structure of the cell surface binding protein [24]. Direct energy minimizations using a constrained Rosetta are used to create the protein structure in this tool. A deep neural network is initially used to predict the interresidue geometries, including distance and orientations, using the input of a protein's amino acid sequence. In the context of Rosetta, the predicted geometries are subsequently turned into restrictions to steer the structure prediction based on direct energy minimization [25]. To further increase the stability of the structure, the interresidual binding energy was minimized using the YASARA energy minimization server [26]. By enhancing physical realism, stereochemistry, and side-chain correctness in the 3D model, the YASARA energy minimization server can aid the homology modelling. ERRAT [27] and Verify3D [28] of the SAVES server and the Ramachandran plot [29] and QMEAN *Z*-scores [30, 31] from the Swiss model were used to validate the projected model's quality. The final 3D model of the protein was captured with the PyMOL2 software [32].

### 2.4. B Cell Epitope Prediction

By extrapolating B cell epitopes from antigens, we can better comprehend the immunological principles underlying the identification of an antigen by an antibody. Additionally, it is crucial for designing vaccines and medications. Utilizing the ABCpred server, the antigens were subjected to linear B cell epitope prediction (http://www.imtech.res.in/raghava/abcpred/) [33]. Based on a recurrent neural network with a threshold value of 0.51, 16-mers was chosen as the preferred window length. The predicted epitopes' antigenicity, allergenicity, and toxicity indexes were evaluated using the VaxiJen v2.0 [34] (threshold value of 0.5), AllerTOP 2.0 [35], and ToxinPred [36] servers, respectively. Moreover, the ability of B cell linear epitopes to induce IFN-*γ* was predicted by the IFNepitope server [37]. Finally, ElliPro (http://tools.iedb.org/ellipro/) [38], an online server, has been used to predict the discontinuous (conformational) B cell epitopes. In this tool, the minimum score for the prediction was set at 0.5, while the maximum distance was set at 6.

### 2.5. Prediction of Cytotoxic T Lymphocyte (CTL) Epitope and MHC I Binding Allele Analysis

Cytotoxic T cell epitope prediction is aimed at identifying the smallest peptides present in an antigen which has the capability to activate T cells or evoke immunogenicity [39]. These antigens have the ability to trigger CD4+ or CD8+ T cells. The CTL epitopes for (9-mers) 12 MHC class I supertypes (A1, A2, A3, A24, A26, B7, B8, B27, B39, B44, B58, and B62) can be predicted by the NetCTL 1.2 server. The selected protein was submitted to the NetCTL v1.2 server (http://www.cbs.dtu.dk/services/NetCTL/) [40] in FASTA format in order to predict the CTL epitope (9-mer). The threshold value was fixed at 0.4 with a sensitivity and specificity of 0.89 and 0.94, respectively. We selected only the A1 supertype within the NetCTL parameters, even though the server can predict CTL epitopes for up to 12 MHC class I supertypes. Information about MHC ligand elution and MHC binding studies can be accessed in the Immune Epitope Database (IEDB), which offers a library of experimentally identified T cell epitopes [41]. The interacting alleles (MHC I-binder) with these epitopes were then revealed using the SMM method by the Immune Epitope Database (IEDB) (http://tools.iedb.org/) [42]. For further study, the MHC-I alleles with the higher affinity (IC50 < 500 nM) for the epitopes were considered.

### 2.6. Epitope Selection for Docking and Epitope Prioritization

Antigenicity is a crucial feature in the development of vaccines. The antigenicity of the peptides was assessed via the VaxiJen 2.0 server (http://www.ddg-pharmfac.net/vaxijen/) with a threshold of 0.4 to verify their capacity to induce an immunological response. The server AllerTOP 2.0 (http://www.ddg-pharmfac.net/AllerTOP/) was utilized to predict allergenicity. It was used to determine whether or not the epitopes were likely to result in allergic reactions in people. Additionally, we used the ToxinPred server (http://crdd.osdd.net/raghava/toxinpred/) to identify the toxicity of each epitope. Moreover, the obtained peptides were then placed into an online program called IFNepitope (http://crdd.osdd.net/raghava/ifnepitope/scan.php) to evaluate the IFN-induction capacity using a hybrid method (motif and SVM) as well as an IFN-*γ* versus non-IFN-*γ* model.

### 2.7. Peptide Designing and Molecular Docking Analysis

APPTEST server [43] was utilized to design the three-dimensional structure of the peptide. With a neural network design and simulated annealing techniques, the APPTEST server can accurately predict the peptide's tertiary structure. The binding interaction between the epitope and receptor molecule was scrutinized by a molecular docking experiment. To perform the docking analysis, the crystal structure of HLA-B^∗^15:01 (PDB ID–1xr8) was first downloaded from the Research Collaboratory for Structural Bioinformatics (RCSB) database [44]. The receptor molecule was subsequently prepared for the docking analysis by removing the associated ligands and water molecules. Subsequently, polar hydrogen molecules were added to the receptor molecule. The docking analysis between the human receptor HLA-B^∗^15:01 and our designed peptide (ligand) was performed by the AutoDock Vina tool [45]. The grid box size of the AutoDock Vina tool was set as 12.702, 31.843, and 18.307, respectively, for *X*, *Y*, and *Z*. Discovery Studio 2021 [46] software was utilized to investigate the binding interactions and residues in the interacting surface between the peptide and receptor. PDBsum [47] was subsequently used for the graphical illustration of the type of interacting bonds.

### 2.8. Molecular Dynamic Simulation

Molecular dynamics is a computer method for describing the molecular behaviors of the receptor and the epitope [48]. The web-based iMODS server (https://imods.iqfr.csic.es/) was utilized for the molecular dynamic simulation investigation to comprehend the residual effects of the vaccine with the receptor. All the parameters in this web tool were kept as default. This website uses NMA in internal coordinates to investigate the collective movements of protein sequences (torsional space) and provides different analyses, including the ability to calculate eigenvalue, *B*-factor, NMA mobility, and deformability [49].

## 3. Results

### 3.1. Sequence Retrieval and Phylogeny Analysis

**Figure 2:**
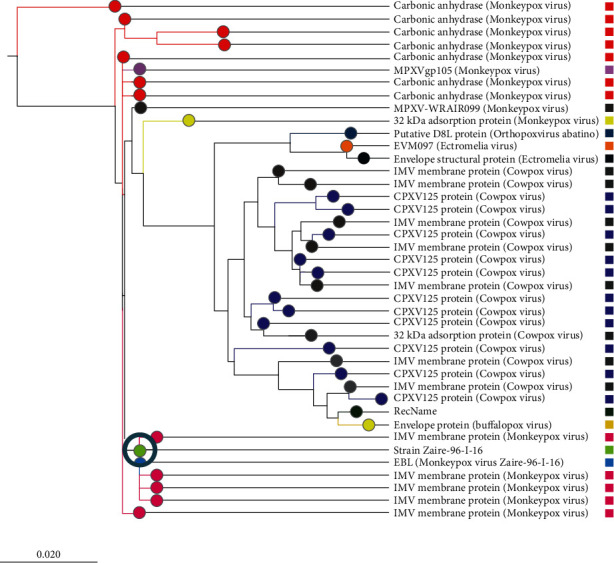
Phylogenetic tree showing the ancestral relationship of our query protein (marked with a circle) with other proteins (scale: 0.020 represents 2% differences between two sequences).

The search result with the keyword “monkeypox cell surface binding protein” in the UniProt database returned 14 protein sequences, four of which were from the Vaccinia virus. Among the ten MPV proteins, the protein with accession Q8V4Y0, which has 304 amino acid residues, was selected for this study as it was marked as reviewed by the UniProt database. By similarity searching, the UniProt database has declared that this sequence is connected with binding to chondroitin sulfate on the cell surface to facilitate the virion attachment to its target cell. As the function of this protein was already verified, it was selected for subsequent analysis. The BLASTp search results against a nonredundant database showed that the query protein shared higher homology with several viruses, including Cowpox, Rabbitpox, Ectromelia, Orthopoxvirus Abatino, Buffalopox, and Vaccinia virus. The analysis of the BLASTp results also showed that the monkeypox virus's cell surface binding protein was homologous to a number of other proteins, including IMV membrane protein, carbonic anhydrase, and CPXV125 protein. A phylogenetic tree was constructed by the neighbor-joining method with the top 40 BLAST sequences (Supplementary Table 2) and is shown in Figure 2.

### 3.2. Homology Modeling, Energy Minimization and Quality Assessment

**Figure 3:**
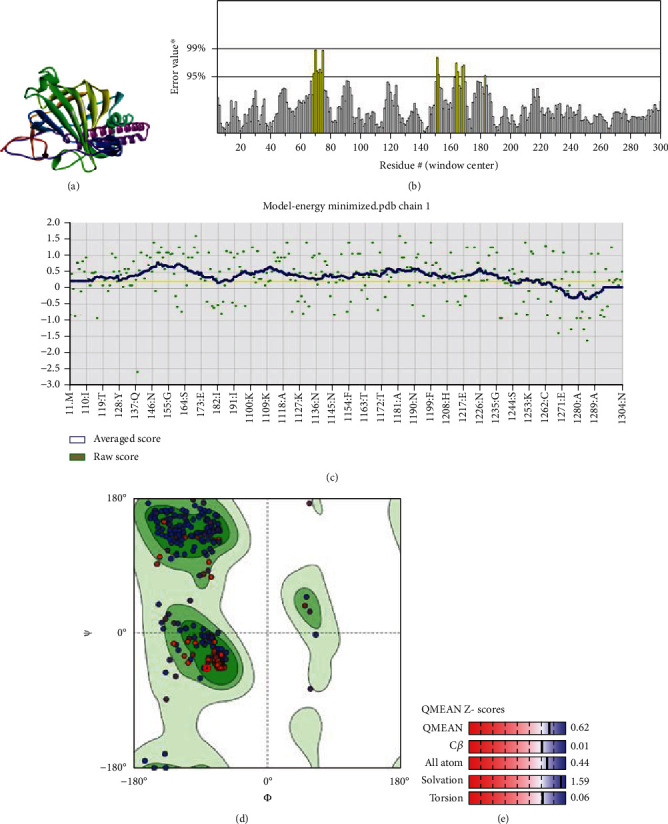
(a) The YASRA energy minimization server-mediated energy-minimized 3D structure of the protein visualized by PyMOL. (b) ERRAT showing an overall quality factor of 95.556; (c) Verify3D showing an overall 3D-1D score ≥ 0.2 for 82.24% of the residues; (d) Swiss model's Ramachandran plot indicating that 95.36% of the residues are in a preferred region; (e) QMEAN *Z*-score chart showing an overall QMEAN score of 0.62.

Recent advancements in structural genomics have made homology modeling a significant aspect of comparative modeling, which is commonly used to predict unknown structures via the use of various tools and databases [50]. When the amino acid sequence is available, homology modeling is an efficient method for predicting protein structure [51]. It has been previously reported in several studies that the 3D structure of a protein and its functional activity are closely linked [52, 53]. Hence, the 3D structure was predicted using the trRosetta server for homology modeling. trRosetta uses its own energy minimization strategy to deliver an accurate structure. Additionally, the YASARA energy minimization server also minimized the energy of the model protein from -126381.6 kJ/mol to -166643.8 kJ/mol to improve the consistency of the protein. A comparison analysis is given in Table 1. After that, the protein's 3D structure was visualized using PyMOL, as shown in Figure 3(a).

However, the refined structure was used to conduct a quality assessment of the modelled protein using the Swiss model and SAVES server. The validity of the predicted model was determined using ERRAT, which assessed the statistics of nonbonded interactions between various atoms based on atomic interactions [54]. According to the ERRAT, the protein structure was found to be of good quality, with a quality factor of 95.556 (Figure 3(b)). The Verify3D server was used to assess an atomic model's (3D) compatibility with its amino acid sequence, and the server ensured the structure with an overall 3D-1D score ≥ 0.2 for 82.24% of the residues (Figure 3(c)), suggesting that the structure was compatible and fairly good. The Swiss model's Ramachandran plot indicated that 95.36% of the residues were in a preferred region (Figure 3(d)). The quality of our model, according to the QMEAN score, which was calculated to be 0.62 considered the predicted model as an excellent model (Figure 3(e)).

### 3.3. Prediction of B Cell Epitope

**Figure 4:**
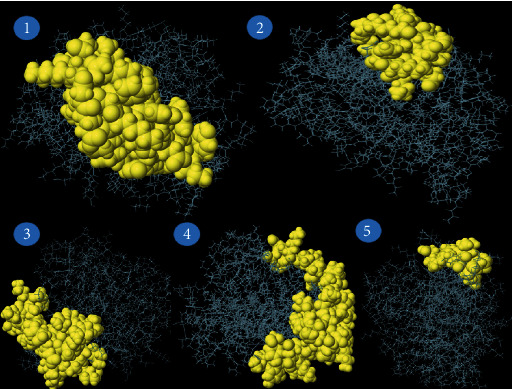
ElliPro tool predicted conformational B cell epitopes. Length and score of each epitope were as follows: (1) 42 residues, score: 0.873; (2) 18 residues, score: 0.682; (3) 32 residues, score: 0.662; (4) 58 residues, score: 0.643; and (5) 12 residues, score: 0.613.

For triggering a humoral immune response, which activates B cells to produce antibodies, B cell epitopes are required. In our study, the ABCpred server predicted 30 peptide sequences of B cell epitopes (Supplementary Table 3). The antigenicity, allergenicity, and toxicity of the selected B cell epitopes were examined. Upon the analysis, we identified 11 peptides to be antigenic, allergenic, and nontoxic among 30 peptide sequences. The peptide QLSPINIETKKAISDT (beginning at position 183) was found to have the highest antigenicity score, and it was 1.206. These 11 B cell epitopes are listed in Table 2. The discontinuous (conformational) B cell epitopes have been predicted using the online server ElliPro. With values ranging from 0.623 to 0.873, five discontinuous B cell epitopes were predicted to have 162 residues and the conformational epitopes were 12 to 58 residues in size (Table 3). ElliPro demonstrated 5 conformational B cell epitopes in the query protein with their lengths, and scores are illustrated in Figure 4.

### 3.4. Prediction of CTL Epitope and Analysis of the MHC I Binding Alleles

The NetCTL 1.2 server predicted a total of 19 cytotoxic T lymphocyte (CTL) epitopes (9-mers) (Supplementary Table 4) according to the MHC A1 supertype but only 11 epitopes were found as antigenic, immunogenic, nonallergenic, and nontoxic among them (Table 4). On the other hand, IFN-*γ* inducing epitopes with anticipated positive and negative results from the IFNepitope server were selected. The MHC-I alleles with higher affinities (IC50 < 500 nM) for the epitopes are listed in Table 4. The 8 antigenic CD8+ T cell epitopes interact with at least one unique MHC-I HLA allele, as reported by the IEDB.

### 3.5. Epitope Selection for Docking and Epitope Prioritization

To be an effective vaccine candidate, an epitope or peptide sequence must fulfil a number of requirements. For example, the epitope must cause an immunogenic response in the host in order to meet the initial condition. Another critical phase of vaccine development is testing for toxicity. An effective vaccine candidate must not produce any toxic response in the host after vaccination. Allergenicity, however, is another significant obstacle to the development of vaccinations. The majority of immunizations produce immunoglobulin E and type 2 helper T (Th2) cells, which stimulate an unwanted allergic immune response [53, 55]. Finally, the design of potential vaccines must ensure that they cause the production of IFN-*γ* following immunization [56]. The majority of vaccines have recently been developed using B cell immunity; however, in this work, we chose to design the vaccine using a T cell epitope since it can result in long-lasting immunity [57]. We initially screened the epitope based on the number of MHC I bindings to find a suitable vaccine candidate. Upon the analysis with IEDB MHC I binding analysis prediction tool, it was found that epitope MSAPFDSVF interacts with the maximum number of alleles. But this epitope was abandoned as the VaxiJen score of this epitope identified the epitope as a nonantigen. Three epitopes, including TTSPVRENY, YVLSTIHIY, and ILFLMSQRY, were found to interact with 4 MHC I binding alleles, and all these epitopes fulfil all other requirements to be an effective candidate for the vaccine. But we selected the epitope “ILFLMSQRY” for further analysis as the VaxiJen-predicted antigenicity score for this epitope was found to be maximum. Four MHC I binding alleles that interacted with the ILFLMSQRY epitope were HLA-B^∗^15:01, HLA-A^∗^30:02, HLA-A^∗^03:01, and HLA-A^∗^32:01. This epitope satisfied each prerequisite that an epitope for vaccination must adhere to. The host's immunogenic response to the epitope was assessed using the VaxiJen 2.0 antigenic analysis tool. The epitope has been identified as a potential antigen after examination with this tool (antigenicity score 1.0469). The epitope was recognized by the ToxinPred server as a nontoxic epitope. The AllerTOP program was used to calculate the sequence-based allergenicity prediction properly, and the predicted query epitope was discovered to be nonallergenic. IFN-*γ* response of our selected epitope was found to be positive (score—0.0499). All these findings have established the epitope as a promising vaccine candidate.

### 3.6. Molecular Docking Analysis

**Figure 5:**
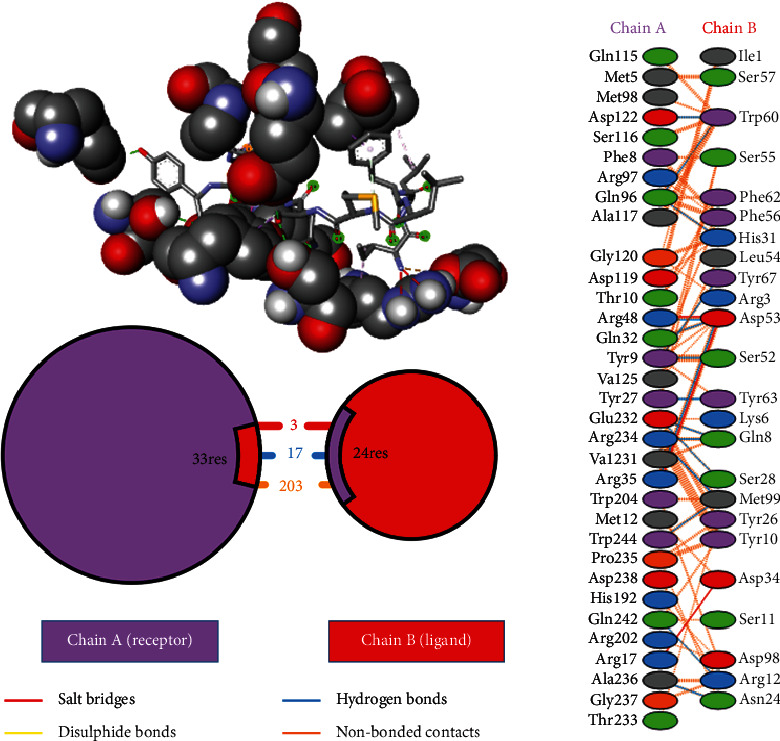
AutoDock Vina-generated docked complex analysis revealed by PDBsum server. Figure represents the graphical illustration of the docked complex and the types of interacting bonds between the peptide “ILFLMSQRY” and receptor HLA-B^∗^15:01.

**Figure 6:**
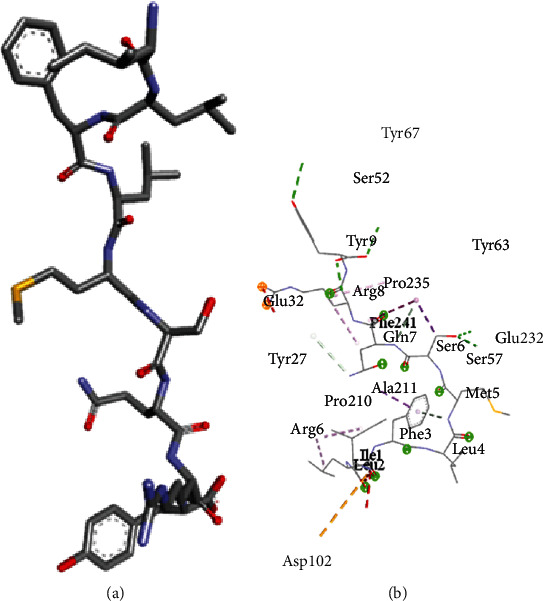
(a) Three-dimensional structure of the predicted epitope, “ILFLMSQRY” and (b) visualization of binding interactions and residues after the docking of “ILFLMSQRY” with HLA-B^∗^15:01.

Ligand-receptor docking has been used to assess the suggested epitope vaccine's affinity for the human leukocyte antigen HLA-B^∗^15:01. Analysis with the AutoDock Vina program has generated 9 binding models between the ligand (epitope) and the receptor (HLA-B^∗^15:01) molecule. The binding energy has been found between the ranges of -7.5 kcal/mol and -6.4 kcal/mol. After some rigorous analysis with PyMOL software, model 1 (illustrated in Figure 5) was identified as the best binding model. In this model, the epitope's binding energy to the HLA-B^∗^15:01 receptor was estimated to be -7.5 kcal/mol. It was also discovered through the docking research that the anticipated epitope formed interactions with 22 amino acid residues, including Tyr67, Ser52, Tyr 63, Tyr9, Pro235, Arg8, Gln32, Phe241, Gln7, Glu232, Ser6, Tyr27, Ser57, Ala211, Met5, Pro210, Arg6, Phe3, Leu4, Ile1, Leu2, and Asp102. The high numbers of interacting bonds and residues and lower energy of the docked complex revealed a stable link between the ligand and the receptor molecule.  Figure 6 displays the Discovery Studio 2021 software-derived three-dimensional structure of the peptide and the binding interactions between the peptide and HLA-B^∗^15:01 following docking analysis. Analysis with PDBsum has revealed 17 hydrogen bonds and 3 salt bridges between the docked complex, which also demonstrated a strong interaction between the receptor and ligand molecule (Figure 5). The interacting hydrogen bonds were TYR27-TYR63, GLN32-ASP53, ARG35-ASP53, ARG48-ASP53, GLN96-HIS31, GLN96-TRP60, ASP122-TRP60, GLU232-GLN8, GLU232-SER28, ARG234-GLN8, ARG234-MET99, ARG234-MET99, ALA236-ASN24, GLN242-ARG12, TRP244-MET99, TYR9-SER52, and TYR9-TYR67, and the chain distances were 3.13, 2.80, 2.61, 3.03, 2.73, 2.74, 2.73, 2.89, 2.54, 2.78, 2.67, 3.26, 2.84, 3.08, 3.12, 3.18, and 2.99, respectively. The graphical illustration of the docked complex and the types of interacting bonds is presented in Figure 5.

### 3.7. Analysis of the Molecular Dynamic Simulation Study

**Figure 7:**
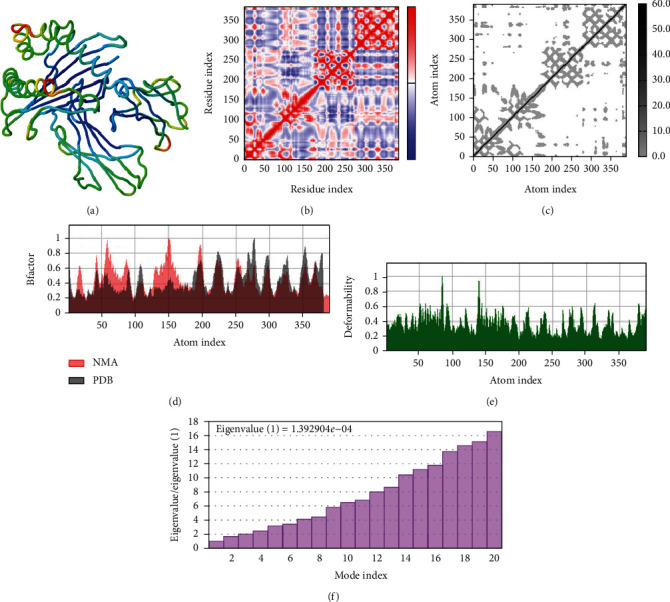
(a) Residual motion of the docked complex. (b) Covariance matrix analysis of the residual pair. (c) Connection spring image of the elastic network model. (d) NMA calculation and PDB *B*-factor. (e) Deformability of the docked complex. (f) Eigenvalue of the docked complex.

A simulation of molecular dynamics was executed to assess the system's stability. The migration of the vaccine construct toward the TLR-4 was confirmed by the estimation of NMA mobility (Figure 7(a)). The decreased fluctuation of the protein residue in the docked complex was substantiated by the NMA calculation and PDB *B*-factor (Figure 7(d)). The elastic network model's connection spring image (Figure 7(c)) revealed the existence of deformability and a few hinges within the residues, reflecting the increased stability of the docked complex (Figure 7(e)). The study of the eigenvalue (Figure 7(f)) and covariance matrix (Figure 7(b)) further supported that the value for deforming the molecular docked complex (1.392904*e*-04) is much greater, indicating that more energy is required to alter the stability of the vaccine-receptor docked complex.

## 4. Discussion

The development of mutant strains of microbial pathogens, changes in host lifestyles, and surrounding environments of the host and pathogen contribute to emerging and reemerging diseases. Some of these diseases are now spreading over the entire world due to increased global trade and tourism rather than being confined to the originating countries [58]. The monkeypox virus has recently started to spread significantly in nonendemic regions. Therefore, taking the required precautions to stop this disease in its early stages is imperative. Vaccines are important preventive measures against life-threatening diseases. The development of computational tools and databases has paved the path to vaccine creation. The conventional methods for vaccine creation which are very time-consuming and costly are now being replaced by more straightforward and cost-effective computational methods [53]. In this study, a computer-based immunoinformatics approach was employed to construct an epitope-based vaccine against the cell surface binding protein of reemerging mPox virus. The viral protein sequence was first obtained from the UniProt databases in FASTA format and subjected to the NCBI protein-protein BLAST program for phylogenetic characterization. Before developing a vaccine, some researchers contend that it could be advantageous to understand more about the virus. Consequently, the phylogenetic study of a particular virus is important because it offers details on a number of areas in viral exploration, including evolutionary relationships and epidemiology [59]. Since the phylogenetic tree may identify common ancestors, it can aid in the development of vaccines if the outbreak is caused by the same species as specified in the phylogenetic tree and also lower the incidence of newly emerging illnesses. As a result, this study is aimed at investigating the genetic or evolutionary associations of the mPox virus with the use of a phylogenetic tree. The findings of the BLASTp search revealed that various viruses, including the Vaccinia virus, Cowpox, Rabbitpox, Ectromelia, Orthopoxvirus Abatino, and Buffalopox, shared higher homology with the studied protein.

The three-dimensional structure of the protein was designed by the trRosetta server, and several other tools verified the quality of the predicted model. The total quality factor of the ERRAT value was 95.556, which is within the acceptable range; values that are lower than 95% are regarded as rejected. More than 90% of residues in the most favored region is considered satisfactory values for the Ramachandran plot. In our study, the Ramachandran plot value was 95.36% for the predicted structure, indicating our structure is perfect. In the Verify 3D plot, 82.24% of the residues got a 3D-1D score greater than 0.2. This value is also higher than the satisfactory value of the Verify3D program, which is considered as 80%. The model's QMEAN *Z*-score was 0.62, very near to 0 and indicating high model quality.

A critical step in the creation of vaccines is the prediction of T and B cell epitopes, which was covered in the current study. According to the investigation, 30 B cell and 19 T cell epitopes were identified in the monkeypox virus's cell surface binding protein. Following the epitope identification, an ideal T cell epitope was taken into consideration for designing the vaccine since it can produce long-lasting protection. Since only antigenic peptides can trigger the immune response in the host, predicting the antigenicity of all protein sequences is an important aspect of vaccine development [60, 61]. Candidates for vaccines have to possess antigenic characteristics [62]. The T cell epitope “ILFLMSQRY” was suggested as the best vaccine candidate in this study based on the antigenicity score, nonallergenic and nontoxic qualities. In our study, the antigenicity of the vaccine candidate was predicted by the VaxiJen 2.0 server to be 1.0469 which is significantly higher than the vaccine candidate studied by Khan et al. [61] and Abdi et al. [63]. Antigenic scores of the selected 3 epitopes for vaccine designing according to the *in silico* study of Khan et al. [61] were reported to be 0.52, 0.56, and 0.76, respectively. The constructed vaccine studied by Abdi et al. [63] had an antigenicity score of 0.685. This epitope was found to interact with 4 MHC I molecules including HLA-B^∗^15:01, HLA-A^∗^30:02, HLA-A^∗^03:01 and HLA-A^∗^32:01.

Immune receptor molecules and antigenic molecules must interact for an immune response to be effectively activated. Therefore, molecular docking between immune receptor molecules and the predicted epitopes was performed to examine potential interactions, binding energy, and configurations. The lower value (negative score) of binding energy demonstrates the characteristics of the high-binding interaction of the constructed vaccine. The docking analysis in this study indicates significant interactions between the ligand and receptor (HLA-B^∗^15:01) with lower binding energy. Additionally, a considerable number of hydrogen bonds (17) and interfering amino acid residues (22) were also present in the docked complex. The binding energy of the epitope to the HLA-B^∗^15:01 receptor was found to be -7.5 kcal/mol as per our study, which is lower than the binding energy of YVLSTIHIY to HLA-DRB1 (−7.1 kcal/mole) according to the *in silico* analysis of Shantier et al. [13]. After molecular docking, a molecular dynamic simulation study was performed to determine the receptor and epitope's molecular characteristics. The NMA mobility, deformability, *B*-factor, eigenvalues, covariance map, and elastic network further confirmed the stability of the docked complex.

## 5. Conclusion

The administration of medications and vaccinations shield people from significant illness and disease-related consequences. So, to find a reliable means of stopping monkeypox virus-associated illness, we have used a bioinformatics technique. Regarding the antigenicity score and nonallergenic and nontoxic properties, our study has identified “QLSPINIETKKAISDT” and “ILFLMSQRY' as the best B cell and T cell epitopes, respectively. This *in silico* study has also suggested that our proposed T cell epitope “ILFLMSQRY” had higher affinity for binding with the receptor of its target and might trigger a potent immune response and function as a therapeutic agent against the monkeypox virus infection. Our study revealed that this highly immunogenic and nonallergenic epitope can effectively interact with the human leukocyte antigen HLA-B^∗^15:01. However, though computational analysis is not always sufficient to provide accurate results, these findings can be further validated through additional research, including *in vitro* characterization and analysis in the lab, followed by *in vivo* work in an animal model.

## Figures and Tables

**Table 1:** Quality assessment score before and after energy minimization.

Parameter	Before energy minimization	After energy minimization
Energy	-126381.6 kJ/mol	-166643.8 kJ/mol
ERRAT quality score	93.5811	95.270
Verify3D score	67.11	82.24
Ramachandran favored	95.36	95.36
QMEAN *Z*-scores	0.74 ± 0.05	0.72 ± 0.05

**Table 2:** Predicted antigenic B cell epitopes found in the protein with their rank, start position, ABCpred score, VaxiJen scores, allergenicity, toxic properties, and IFN-*γ* responses.

Sequence	Start position	Score	Antigenicity	Allergenicity	Toxicity	IFN-*γ* response
YSSYEEAKKHDDGIII	101	0.87	0.4193). (probable antigen)	Nonallergen	Nontoxin	Negative -0.46835004
KPHYITENYRNPYKLN	211	0.84	0.6352 (probable antigen)	Nonallergen	Nontoxin	Negative -1.1321044
KTFAIIAIVFVFILTA	274	0.81	0.7642 (probable antigen)	Nonallergen	Nontoxin	Positive 0.66523392
GTTINHSADAAWIIFP	171	0.72	0.6797 (probable antigen)	Nonallergen	Nontoxin	Negative -0.32073495
LDSIRSANMSAPFDSV	138	0.71	0.5628 (probable antigen)	Nonallergen	Nontoxin	Negative -0.064118698
GFLPNEYVLSTIHIYW	55	0.70	0.8244 (probable antigen)	Nonallergen	Nontoxin	Negative -0.20292443
LVRINFKGGYISGGFL	42	0.68	1.1759 (probable antigen)	Nonallergen	Nontoxin	Negative -0.51981652
QLSPINIETKKAISDT	4	0.68	1.2064 (probable antigen)	Nonallergen	Nontoxin	Negative -0.66219986
GKEDDYGSNHLIDVYK	71	0.60	0.4631 (probable antigen)	Nonallergen	Nontoxin	Positive 0.1582591
IHYNESKPTTIQNTGK	26	0.59	0.5888 (probable antigen)	Nonallergen	Nontoxin	Negative -0.34832699
AILFLMSQRYSREKQN	289	0.54	0.5770 (probable antigen)	Nonallergen	Nontoxin	Negative -0.32788544

**Table 3:** Predicted conformational B cell epitopes.

No.	Residues	No. of residues	Score
1	A:Y250, A:F251, A:M252, A:K253, A:W254, A:L255, A:S256, A:D257, A:L258, A:R259, A:E260, A:A261, A:C262, A:F263, A:S264, A:Y265, A:Y266, A:Q267, A:K268, A:Y269, A:I270, A:E271, A:G272, A:N273, A:K274, A:T275, A:F276, A:A277, A:I278, A:I279, A:A280, A:I281, A:V282, A:F283, A:V284, A:F285, A:I286, A:L287, A:T288, A:A289, A:I290, A:L291	42	0.873

2	A:L57, A:P58, A:N59, A:E60, A:N97, A:K98, A:K99, A:K100, A:Y101, A:S102, A:S103, A:E105, A:E106, A:A107, A:K108, A:K109, A:H110, A:G113	18	0.682

3	A:M1, A:P2, A:Q3, A:Q4, A:L5, A:E11, A:T12, A:K13, A:K14, A:A15, A:I16, A:S17, A:D18, A:T19, A:R20, A:L21, A:K22, A:T23, A:G55, A:L81, A:I82, A:D83, A:V84, A:K224, A:L225, A:N226, A:D227, A:D228, A:T229, A:Q230, A:V231, A:Y232	32	0.662

4	A:N29, A:E30, A:S31, A:K32, A:T34, A:T35, A:T39, A:G40, A:K41, A:K48, A:G49, A:G50, A:G71, A:K72, A:E73, A:D74, A:D75, A:Y76, A:K86, A:N136, A:Q137, A:D139, A:S140, A:I141, A:R142, A:S143, A:A144, A:N145, A:M146, A:S147, A:A148, A:P149, A:F150, A:D151, A:S152, A:V153, A:D194, A:S197, A:K198, A:R200, A:T201, A:L202, A:L203, A:S204, A:S205, A:S206, A:N207, A:H208, A:E209, A:G210, A:K211, A:P212, A:H213, A:Y214, A:I215, A:T216, A:E217, A:Y219	58	0.643

5	A:E236, A:I237, A:I238, A:A240, A:A241, A:T243, A:S244, A:P245, A:V246, A:R247, A:E248, A:N249	12	0.613

**Table 4:** Antigenic CTL epitopes predicted with NetCTL 1.2 with their combined score, interacting alleles, VaxiJen scores, allergenicity, toxic properties, and IFN-*γ* responses.

Epitopes	Combined score	Interacting MHC I alleles (IC50 < 500 nm)	Number of interacting alleles	Antigenicity	Allergenicity	Toxicity	IFN-*γ* response
ITENYRNPY	3.0880	HLA-A^∗^01:01, HLA-A^∗^30:02	2	0.8011 (probable antigen)	Nonallergen	Nontoxin	Negative -0.73284923
TTSPVRENY	2.7080	HLA-A^∗^68:01, HLA-B^∗^58:01, HLA-A^∗^11:01, HLA-A^∗^30:02	4	0.7917 (probable antigen)	Nonallergen	Nontoxin	Negative -0.33212602
VSDHKNVYF	1.8846	HLA-A^∗^01:01	1	1.0903 (probable antigen)	Nonallergen	Nontoxin	Negative -0.79520714
YVLSTIHIY	1.3997	HLA-B^∗^35:01, HLA-A^∗^30:02,zHLA-B^∗^15:01, HLA-A^∗^26:01	4	0.5976 (probable antigen)	Nonallergen	Nontoxin	Negative -0.11773186
SADAAWIIF	1.1852	HLA-B^∗^35:01	1	0.8244 (probable antigen)	Nonallergen	Nontoxin	Positive 0.0055844258
HSADAAWII	1.1798	HLA-B^∗^58:01, HLA-A^∗^68:02, HLA-A^∗^02:06	3	0.8212 (probable antigen)	Nonallergen	Nontoxin	Positive 0.081548412
RLKTLDIHY	0.9206	HLA-B^∗^15:01, HLA-A^∗^30:02, HLA-A^∗^30:01	3	1.9035 (probable antigen)	Nonallergen	Nontoxin	Positive 0.35168917
LSDLREACF	0.9079	None	0	1.7255 (probable antigen)	Nonallergen	Nontoxin	Negative -0.42189004
LREACFSYY	0.8316	None	0	1.5067 (probable antigen)	Nonallergen	Nontoxin	Negative -0.21162008
YSGEINLVH	0.8202	None	0	0.8764 (probable antigen)	Nonallergen	Nontoxin	Negative -0.72581695
ILFLMSQRY	0.7904	HLA-B^∗^15:01, HLA-A^∗^30:02, HLA-A^∗^03:01, HLA-A^∗^32:01	4	1.0469 (probable antigen)	Nonallergen	Nontoxin	Positive 0.049915841

## Data Availability

The dataset(s) supporting the conclusions of this article is (are) included within the article.

## Abbreviations

3D: Three-dimensional
CTL: Cytotoxic T lymphocytes
HLA: Human leukocyte antigen
IEDB: Immune Epitope Database
IFN-*γ*: Interferon-gamma
MHC I: Major histocompatibility complex I
MPV: Monkeypox virus
mPox: Monkeypox
NCBI: National Center for Biotechnology Information
RCSB: Research Collaboratory for Structural Bioinformatics
SARS-CoV-2: Severe acute respiratory syndrome-corona virus-2
SMM: Stabilized matrix method
Th2: Helper T cell 2.

## Supplementary Materials

Supplementary Materials Supplementary Table 1: list of bioinformatics tools and databases employed in this study. Supplementary Table 2: the top 41 protein sequences (including the query protein) retrieved from the BLASTp search from the NCBI. Supplementary Table 3: predicted B cell epitopes found in the protein with their rank, start position, ABCpred score, VaxiJen scores, allergenicity, toxic properties, and IFN-*γ* responses. Supplementary Table 4: predicted T cell epitopes found in the protein with their combined score, interacting alleles, VaxiJen scores, allergenicity, toxic properties, and IFN-*γ* responses.
