# Supplementary material for: Identification of B and T Cell Epitopes to Design an Epitope-Based Peptide Vaccine against the Cell Surface Binding Protein of Monkeypox Virus: An Immunoinformatics Study
Source: J Immunol Res. 2023 Feb 22;2023:2274415. doi: 10.1155/2023/2274415 (PMC9977553; doi:10.1155/2023/2274415)
Supplement: Supplementary Materials — Supplementary Table 1: list of bioinformatics tools and databases employed in this study. Supplementary Table 2: the top 41 protein sequences (including the query protein) retrieved from the BLASTp search from the NCBI. Supplementary Table 3: predicted B cell epitopes found in the protein with their rank, start position, ABCpred score, VaxiJen scores, allergenicity, toxic properties, and IFN-γ responses. Supplementary Table 4: predicted T cell epitopes found in the protein with their combined score, interacting alleles, VaxiJen scores, allergenicity, toxic properties, and IFN-γ responses. [file 2274415.f1.docx]

**Supplementary files**

**Supplementary File 1:**

>sp|Q8V4Y0|CAHH_MONPZ Cell surface-binding protein OS=Monkeypox virus (strain Zaire-96-I-16) OX=619591 GN=E8L PE=2 SV=1

MPQQLSPINIETKKAISDTRLKTLDIHYNESKPTTIQNTGKLVRINFKGGYISGGFLPNEYVLSTIHIYWGKEDDYGSNHLIDVYKYSGEINLVHWNKKKYSSYEEAKKHDDGIIIIAIFLQVSDHKNVYFQKIVNQLDSIRSANMSAPFDSVFYLDNLLPSTLDYFTYLGTTINHSADAAWIIFPTPINIHSDQLSKFRTLLSSSNHEGKPHYITENYRNPYKLNDDTQVYYSGEIIRAATTSPVRENYFMKWLSDLREACFSYYQKYIEGNKTFAIIAIVFVFILTAILFLMSQRYSREKQN

**Supplementary Table 1:** List of bioinformatics tools and databases employed in this study

| **S. N** | **Tools name** | **Mechanism** | **References** |
| --- | --- | --- | --- |
| 01. | UniProt database | To retrieve sequence | [21] |
| 02 | BLASTp | To Find similar sequences in protein Databases and construct a phylogenetic tree | [22] |
| 03 | Microreact database | To visualize a phylogenetic tree | [23] |
| 04 | trRosetta | To predicts protein structures and construct the three-dimensional structure | [24] |
| 05 | YASARA energy minimization server | To further increase the stability of the structure | [26] |
| 06 | ERRAT | To analyze the statistics of nonbonded interactions between different atoms and verify protein structures | [27] |
| 07 | Verify3D | To verify the structure | [28] |
| 08 | Ramachandran plot | To evaluate the stability of the predicted structures of protein molecules. | [29] |
| 09 | QMEAN Z-scores | To verify the three-dimensional structure of the protein | [30, 31] |
| 10 | PyMOL2 software | To visualize the 3D structure of the protein | [32] |
| 11 | ABCpred server | To predict the linear B-cell epitopes | [33] |
| 12 | VaxiJen v2.0 | To assess the antigenicity of the revealed B cell and T cell epitopes | [34] |
| 13 | AllerTOP 2.0 | To predict the antigenicity of the epitopes | [35] |
| 14 | ToxinPred | To identify the toxicity index of each epitope | [36] |
| 15 | IFNepitope server | To anticipate and design IFN-gamma-inducing peptides | [37] |
| 16 | ElliPro | To predict the discontinuous B-cell epitopes. | [38] |
| 17 | NetCTL 1.2 server | To predict the CTL epitopes | [40] |
| 18 | Immune Epitope Database (IEDB) | To predict the MHC I binding alleles | [41] |
| 19 | APPTEST server | To design the three-dimensional structure of the peptide | [43] |
| 20 | The Research Collaboratory for Structural Bioinformatics (RCSB) database | To retrieve the receptor molecule | [44] |
| 21 | AutoDockVina tool | To perform the docking analysis | [45] |
| 22 | Discovery Studio 2021 | To investigate the binding interactions and residues in the interacting surface between the peptide and receptor. | [46] |
| 23 | PDBsum | To retrieve the graphical illustration of the type of interacting bonds. | [47] |
| 24 | iMods | To perform molecular dynamic simulation | [49] |

**Supplementary Table 2:** The top 41 protein sequences (including the query protein) retrieved from the BlastP search from the NCBI.

| Description | Scientific Name | Max Score | Total Score | Query Cover | E value | Per. ident | Acc. Len | Accession |
| --- | --- | --- | --- | --- | --- | --- | --- | --- |
| E8L [Monkeypox virus Zaire-96-I-16] | Monkeypox virus Zaire-96-I-16 | 564 | 564 | 100% | 0 | 100 | 304 | [NP_536532.1](https://www.ncbi.nlm.nih.gov/protein/NP_536532.1?report=genbank&log$=prottop&blast_rank=1&RID=GJ42S22A013) |
| IMV membrane protein [Monkeypox virus] | Monkeypox virus | 564 | 564 | 100% | 0 | 99.67 | 304 | [ADK39130.1](https://www.ncbi.nlm.nih.gov/protein/ADK39130.1?report=genbank&log$=prottop&blast_rank=2&RID=GJ42S22A013) |
| IMV membrane protein [Monkeypox virus] | Monkeypox virus | 563 | 563 | 100% | 0 | 99.67 | 304 | [ADX22948.1](https://www.ncbi.nlm.nih.gov/protein/ADX22948.1?report=genbank&log$=prottop&blast_rank=3&RID=GJ42S22A013) |
| IMV membrane protein [Monkeypox virus] | Monkeypox virus | 563 | 563 | 100% | 0 | 99.67 | 304 | [QNI38872.1](https://www.ncbi.nlm.nih.gov/protein/QNI38872.1?report=genbank&log$=prottop&blast_rank=4&RID=GJ42S22A013) |
| Carbonic anhydrase [Monkeypox virus] | Monkeypox virus | 562 | 562 | 100% | 0 | 99.67 | 304 | [YP_010377102.1](https://www.ncbi.nlm.nih.gov/protein/YP_010377102.1?report=genbank&log$=prottop&blast_rank=5&RID=GJ42S22A013) |
| IMV membrane protein [Monkeypox virus] | Monkeypox virus | 561 | 561 | 100% | 0 | 99.67 | 304 | [AAY97104.1](https://www.ncbi.nlm.nih.gov/protein/AAY97104.1?report=genbank&log$=prottop&blast_rank=6&RID=GJ42S22A013) |
| MPXV-WRAIR099 [Monkeypox virus] | Monkeypox virus | 561 | 561 | 100% | 0 | 99.34 | 304 | [AAU01309.1](https://www.ncbi.nlm.nih.gov/protein/AAU01309.1?report=genbank&log$=prottop&blast_rank=7&RID=GJ42S22A013) |
| IMV membrane protein [Monkeypox virus] | Monkeypox virus | 560 | 560 | 100% | 0 | 99.34 | 304 | [AUW64186.1](https://www.ncbi.nlm.nih.gov/protein/AUW64186.1?report=genbank&log$=prottop&blast_rank=8&RID=GJ42S22A013) |
| MPXVgp105 [Monkeypox virus] | Monkeypox virus | 560 | 560 | 100% | 0 | 99.34 | 304 | [USC26160.1](https://www.ncbi.nlm.nih.gov/protein/USC26160.1?report=genbank&log$=prottop&blast_rank=9&RID=GJ42S22A013) |
| Carbonic anhydrase [Monkeypox virus] | Monkeypox virus | 560 | 560 | 100% | 0 | 99.34 | 304 | [UUV53173.1](https://www.ncbi.nlm.nih.gov/protein/UUV53173.1?report=genbank&log$=prottop&blast_rank=10&RID=GJ42S22A013) |
| Carbonic anhydrase [Monkeypox virus] | Monkeypox virus | 560 | 560 | 100% | 0 | 99.34 | 304 | [UUV60511.1](https://www.ncbi.nlm.nih.gov/protein/UUV60511.1?report=genbank&log$=prottop&blast_rank=11&RID=GJ42S22A013) |
| Carbonic anhydrase [Monkeypox virus] | Monkeypox virus | 559 | 559 | 100% | 0 | 99.34 | 304 | [UUV50130.1](https://www.ncbi.nlm.nih.gov/protein/UUV50130.1?report=genbank&log$=prottop&blast_rank=12&RID=GJ42S22A013) |
| 32 kDa adsorption protein [Monkeypox virus] | Monkeypox virus | 555 | 555 | 100% | 0 | 98.36 | 304 | [CAA66449.1](https://www.ncbi.nlm.nih.gov/protein/CAA66449.1?report=genbank&log$=prottop&blast_rank=13&RID=GJ42S22A013) |
| Carbonic anhydrase [Monkeypox virus] | Monkeypox virus | 554 | 554 | 100% | 0 | 97.37 | 304 | [UVE15191.1](https://www.ncbi.nlm.nih.gov/protein/UVE15191.1?report=genbank&log$=prottop&blast_rank=14&RID=GJ42S22A013) |
| CPXV125 protein [Cowpox virus] | Cowpox virus | 547 | 547 | 100% | 0 | 97.04 | 304 | [NP_619909.1](https://www.ncbi.nlm.nih.gov/protein/NP_619909.1?report=genbank&log$=prottop&blast_rank=15&RID=GJ42S22A013) |
| CPXV125 protein [Cowpox virus] | Cowpox virus | 546 | 546 | 100% | 0 | 96.71 | 304 | [ARR30022.1](https://www.ncbi.nlm.nih.gov/protein/ARR30022.1?report=genbank&log$=prottop&blast_rank=16&RID=GJ42S22A013) |
| IMV membrane protein [Cowpox virus] | Cowpox virus | 544 | 544 | 100% | 0 | 96.71 | 304 | [ADZ29448.1](https://www.ncbi.nlm.nih.gov/protein/ADZ29448.1?report=genbank&log$=prottop&blast_rank=17&RID=GJ42S22A013) |
| Carbonic anhydrase [Monkeypox virus] | Monkeypox virus | 544 | 544 | 100% | 0 | 97.37 | 304 | [UVF74404.1](https://www.ncbi.nlm.nih.gov/protein/UVF74404.1?report=genbank&log$=prottop&blast_rank=18&RID=GJ42S22A013) |
| CPXV125 protein [Cowpox virus] | Cowpox virus | 544 | 544 | 100% | 0 | 96.38 | 304 | [ARR29817.1](https://www.ncbi.nlm.nih.gov/protein/ARR29817.1?report=genbank&log$=prottop&blast_rank=19&RID=GJ42S22A013) |
| Carbonic anhydrase [Monkeypox virus] | Monkeypox virus | 543 | 543 | 100% | 0 | 95.72 | 304 | [UVB81831.1](https://www.ncbi.nlm.nih.gov/protein/UVB81831.1?report=genbank&log$=prottop&blast_rank=20&RID=GJ42S22A013) |
| EVM097 [Ectromelia virus] | Ectromelia virus | 542 | 542 | 100% | 0 | 95.72 | 304 | [NP_671615.1](https://www.ncbi.nlm.nih.gov/protein/NP_671615.1?report=genbank&log$=prottop&blast_rank=21&RID=GJ42S22A013) |
| CPXV125 protein [Cowpox virus] | Cowpox virus | 542 | 542 | 100% | 0 | 96.05 | 304 | [ATB55167.1](https://www.ncbi.nlm.nih.gov/protein/ATB55167.1?report=genbank&log$=prottop&blast_rank=22&RID=GJ42S22A013) |
| IMV membrane protein [Cowpox virus] | Cowpox virus | 541 | 541 | 100% | 0 | 96.05 | 304 | [ADZ30517.1](https://www.ncbi.nlm.nih.gov/protein/ADZ30517.1?report=genbank&log$=prottop&blast_rank=23&RID=GJ42S22A013) |
| IMV membrane protein [Cowpox virus] | Cowpox virus | 541 | 541 | 100% | 0 | 95.39 | 304 | [ADW95400.1](https://www.ncbi.nlm.nih.gov/protein/ADW95400.1?report=genbank&log$=prottop&blast_rank=24&RID=GJ42S22A013) |
| RecName: Full=Cell surface-binding protein; AltName: Full=Carbonic anhydrase homolog [Rabbitpox virus Utrecht] | Rabbitpox virus Utrecht | 541 | 541 | 100% | 0 | 95.39 | 304 | [Q6RZI9.1](https://www.ncbi.nlm.nih.gov/protein/Q6RZI9.1?report=genbank&log$=prottop&blast_rank=25&RID=GJ42S22A013) |
| CPXV125 protein [Cowpox virus] | Cowpox virus | 541 | 541 | 100% | 0 | 95.72 | 304 | [ARR31030.1](https://www.ncbi.nlm.nih.gov/protein/ARR31030.1?report=genbank&log$=prottop&blast_rank=26&RID=GJ42S22A013) |
| 32 kDa adsorption protein [Cowpox virus] | Cowpox virus | 540 | 540 | 100% | 0 | 96.05 | 304 | [CAA66452.1](https://www.ncbi.nlm.nih.gov/protein/CAA66452.1?report=genbank&log$=prottop&blast_rank=27&RID=GJ42S22A013) |
| CPXV125 protein [Cowpox virus] | Cowpox virus | 540 | 540 | 100% | 0 | 95.39 | 304 | [AGY97317.1](https://www.ncbi.nlm.nih.gov/protein/AGY97317.1?report=genbank&log$=prottop&blast_rank=28&RID=GJ42S22A013) |
| putative D8L protein [Orthopoxvirus Abatino] | Orthopoxvirus Abatino | 540 | 540 | 100% | 0 | 95.39 | 304 | [YP_010085781.1](https://www.ncbi.nlm.nih.gov/protein/YP_010085781.1?report=genbank&log$=prottop&blast_rank=29&RID=GJ42S22A013) |
| IMV membrane protein [Cowpox virus] | Cowpox virus | 540 | 540 | 100% | 0 | 95.39 | 304 | [ADZ29663.1](https://www.ncbi.nlm.nih.gov/protein/ADZ29663.1?report=genbank&log$=prottop&blast_rank=30&RID=GJ42S22A013) |
| envelope structural protein [Ectromelia virus] | Ectromelia virus | 540 | 540 | 100% | 0 | 95.39 | 304 | [CAG7620781.1](https://www.ncbi.nlm.nih.gov/protein/CAG7620781.1?report=genbank&log$=prottop&blast_rank=31&RID=GJ42S22A013) |
| CPXV125 protein [Cowpox virus] | Cowpox virus | 540 | 540 | 100% | 0 | 95.72 | 304 | [ARR30190.1](https://www.ncbi.nlm.nih.gov/protein/ARR30190.1?report=genbank&log$=prottop&blast_rank=32&RID=GJ42S22A013) |
| IMV membrane protein [Cowpox virus] | Cowpox virus | 540 | 540 | 100% | 0 | 95.72 | 304 | [ABD97464.1](https://www.ncbi.nlm.nih.gov/protein/ABD97464.1?report=genbank&log$=prottop&blast_rank=33&RID=GJ42S22A013) |
| CPXV125 protein [Cowpox virus] | Cowpox virus | 540 | 540 | 100% | 0 | 95.72 | 304 | [AGY98826.1](https://www.ncbi.nlm.nih.gov/protein/AGY98826.1?report=genbank&log$=prottop&blast_rank=34&RID=GJ42S22A013) |
| envelope protein [Buffalopox virus] | Buffalopox virus | 540 | 540 | 100% | 0 | 95.07 | 304 | [ABC86842.1](https://www.ncbi.nlm.nih.gov/protein/ABC86842.1?report=genbank&log$=prottop&blast_rank=35&RID=GJ42S22A013) |
| CPXV125 protein [Cowpox virus] | Cowpox virus | 540 | 540 | 100% | 0 | 94.74 | 304 | [AGY99251.1](https://www.ncbi.nlm.nih.gov/protein/AGY99251.1?report=genbank&log$=prottop&blast_rank=36&RID=GJ42S22A013) |
| IMV membrane protein [Cowpox virus] | Cowpox virus | 540 | 540 | 100% | 0 | 95.72 | 304 | [ARB50347.1](https://www.ncbi.nlm.nih.gov/protein/ARB50347.1?report=genbank&log$=prottop&blast_rank=37&RID=GJ42S22A013) |
| CPXV125 protein [Cowpox virus] | Cowpox virus | 539 | 539 | 100% | 0 | 95.39 | 304 | [AGY98180.1](https://www.ncbi.nlm.nih.gov/protein/AGY98180.1?report=genbank&log$=prottop&blast_rank=38&RID=GJ42S22A013) |
| CPXV125 protein [Cowpox virus] | Cowpox virus | 539 | 539 | 100% | 0 | 95.39 | 304 | [QEM25205.1](https://www.ncbi.nlm.nih.gov/protein/QEM25205.1?report=genbank&log$=prottop&blast_rank=39&RID=GJ42S22A013) |
| IMV membrane protein [Cowpox virus] | Cowpox virus | 539 | 539 | 100% | 0 | 95.39 | 304 | [AQQ12981.1](https://www.ncbi.nlm.nih.gov/protein/AQQ12981.1?report=genbank&log$=prottop&blast_rank=40&RID=GJ42S22A013) |

**Supplementary Table 3:** Predicted B-cell epitopes found in the protein with their rank, start position, ABCpred score, VaxiJen scores, allergenicity, toxic properties, and IFN-γ responses

| Rank | Sequence | Start position | Score | Antigenicity | Allergenicity | Toxicity | IFN-γ response |
| --- | --- | --- | --- | --- | --- | --- | --- |
| 1 | TIHIYWGKEDDYGSNH | 65 | 0.97 | 1.1624 (Probable antigen) | Allergen | Non-Toxin | POSITIVE 0.13992648 |
| 2 | GEIIRAATTSPVRENY | 235 | 0.92 | 0.2688 (Probable non-antigen) | Non-allergen | Non-Toxin | POSITIVE 0.88963758 |
| 3 | GGYISGGFLPNEYVLS | 49 | 0.91 | 0.3077 (Probable non-antigen) | Non-allergen | Non-Toxin | NEGATIVE  -0.23286766 |
| 4 | YSSYEEAKKHDDGIII | 101 | 0.87 | 0.4193) (Probable antigen) | Non-allergen | Non-Toxin | NEGATIVE  -0.46835004 |
| 5 | FSYYQKYIEGNKTFAI | 263 | 0.86 | 0.2938 (Probable non-antigen) | Allergen | Non-Toxin | NEGATIVE -0.06469366 |
| 6 | NHLIDVYKYSGEINLV | 79 | 0.85 | 0.0755 (Probable non-antigen) | Allergen | Non-Toxin | NEGATIVE -0.50472924 |
| 7 | KPHYITENYRNPYKLN | 211 | 0.84 | 0.6352 (Probable antigen) | Non-allergen | Non-Toxin | NEGATIVE -1.1321044 |
| 7 | DAAWIIFPTPINIHSD | 179 | 0.84 | 0.3628).(Probable non-antigen) | Allergen | Non-Toxin | POSITIVE 0.20944457 |
| 8 | TTSPVRENYFMKWLSD | 242 | 0.82 | 0.3638 (Probable non-antigen) | Non-allergen | Non-Toxin | NEGATIVE -0.39375426 |
| 9 | KTFAIIAIVFVFILTA | 274 | 0.81 | 0.7642) (Probable antigen) | Non-allergen | Non-Toxin | POSITIVE 0.66523392 |
| 9 | QVSDHKNVYFQKIVNQ | 122 | 0.81 | 0.2938 (Probable non-antigen) | Non-allergen | Non-Toxin | NEGATIVE -0.87016298 |
| 10 | PSTLDYFTYLGTTINH | 161 | 0.80 | 0.7620 (Probable antigen) | Allergen | Non-Toxin | NEGATIVE -0.058775127 |
| 11 | YKLNDDTQVYYSGEII | 223 | 0.79 | 0.0312 (Probable non-antigen) | Non-allergen | Non-Toxin | NEGATIVE -0.52939146 |
| 12 | MSAPFDSVFYLDNLLP | 146 | 0.78 | 0.2032 (Probable non-antigen) | Allergen | Non-Toxin | NEGATIVE -0.25867292 |
| 13 | FQKIVNQLDSIRSANM | 131 | 0.75 | 0.1612 (Probable non-antigen) | Non-allergen | Non-Toxin | NEGATIVE -0.28539367 |
| 14 | YSGEINLVHWNKKKYS | 87 | 0.74 | 0.9824 (Probable antigen) | Allergen | Non-Toxin | NEGATIVE -0.39149764 |
| 15 | GTTINHSADAAWIIFP | 171 | 0.72 | 0.6797 (Probable antigen) | Non-allergen | Non-Toxin | NEGATIVE -0.32073495 |
| 16 | PTTIQNTGKLVRINFK | 33 | 0.71 | 1.2221 (Probable antigen) | Allergen | Non-Toxin | NEGATIVE -0.062976903 |
| 16 | LDSIRSANMSAPFDSV | 138 | 0.71 | 0.5628 (Probable antigen) | Non-allergen | Non-Toxin | NEGATIVE -0.064118698 |
| 17 | GFLPNEYVLSTIHIYW | 55 | 0.70 | 0.8244 (Probable antigen) | Non-allergen | Non-Toxin | NEGATIVE -0.20292443 |
| 18 | SKFRTLLSSSNHEGKP | 197 | 0.69 | 0.0792 (Probable non-antigen) | Non-allergen | Non-Toxin | NEGATIVE -0.17130608 |
| 19 | LVRINFKGGYISGGFL | 42 | 0.68 | 1.1759 (Probable antigen) | Non-allergen | Non-Toxin | NEGATIVE -0.51981652 |
| 19 | QLSPINIETKKAISDT | 4 | 0.68 | 1.2064 (Probable antigen) | Non-allergen | Non-Toxin | NEGATIVE -0.66219986 |
| 20 | TRLKTLDIHYNESKPT | 19 | 0.67 | 0.9995 (Probable antigen) | Allergen | Non-Toxin | NEGATIVE -0.35787221 |
| 21 | KWLSDLREACFSYYQK | 253 | 0.62 | 0.7967 (Probable antigen) | Allergen | Non-Toxin | NEGATIVE -0.33260261 |
| 22 | DGIIIIAIFLQVSDHK | 112 | 0.61 | 0.2835 (Probable non-antigen) | Non-allergen | Non-Toxin | POSITIVE 0.55353793 |
| 23 | GKEDDYGSNHLIDVYK | 71 | 0.60 | 0.4631 (Probable antigen) | Non-allergen | Non-Toxin | POSITIVE 0.1582591 |
| 24 | IHYNESKPTTIQNTGK | 26 | 0.59 | 0.5888 (Probable antigen) | Non-allergen | Non-Toxin | NEGATIVE -0.34832699 |
| 25 | NIHSDQLSKFRTLLSS | 190 | 0.58 | -0.5756 (Probable non-antigen) | Non-allergen | Non-Toxin | NEGATIVE -0.20480821 |
| 26 | AILFLMSQRYSREKQN | 289 | 0.54 | 0.5770) (Probable antigen) | Non-allergen | Non-Toxin | NEGATIVE -0.32788544 |

**Supplementary Table 4:** Predicted T-cell epitopes found in the protein with their combined score, interacting alleles, VaxiJen scores, allergenicity, toxic properties, and IFN-γ responses

| Epitopes | Combined score | Interacting MHC I alleles (IC50<500nm) | Number of interacting alleles | Antigenicity | Allergenicity | Toxicity | IFN-γ response |
| --- | --- | --- | --- | --- | --- | --- | --- |
| ITENYRNPY | 3.0880 | HLA-A*01:01, HLA-A*30:02 | 2 | 0.8011 (Probable antigen) | Non-allergen | Non-Toxin | NEGATIVE  -0.73284923 |
| TTSPVRENY | 2.7080 | HLA-A*68:01, HLA-B*58:01, HLA-A*11:01, HLA-A*30:02 | 4 | 0.7917 (Probable antigen) | Non-allergen | Non-toxin | NEGATIVE -0.33212602 |
| VSDHKNVYF | 1.8846 | HLA-A*01:01 | 1 | 1.0903 (Probable antigen) | Non-allergen | Non-Toxin | NEGATIVE -0.79520714 |
| GSNHLIDVY | 1.8607 | HLA-B*15:01, HLA-A*30:02, HLA-B*58:01 | 3 | -0.0002 (Probable non-antigen) | Allergen | Non-Toxin | POSITIVE 0.096832792 |
| QVSDHKNVY | 1.5311 | HLA-B*35:01, HLA-B*15:01 | 2 | 0.2986 (Probable non-antigen) | Allergen | Non-Toxin | NEGAT VE -0.66427787 |
| PSTLDYFTY | 1.5010 | None | 0 | 0.2981 (Probable non-antigen) | Non-allergen | Non-Toxin | NEGATIVE -0.0072201292 |
| LNDDTQVYY | 1.4151 | HLA-A*01:01 | 1 | -0.1260 (Probable non-antigen) | Non-allergen | Non-Toxin | NEGATIVE -0.15189967 |
| YVLSTIHIY | 1.3997 | HLA-B*35:01, HLA-A*30:02,  HLA-B*15:01, HLA-A*26:01 | 4 | 0.5976 (Probable antigen) | Non-allergen | Non-toxin | NEGATIVE -0.11773186 |
| MSAPFDSVF | 1.3277 | HLA-B*35:01, HLA-B*58:01, HLA-B*15:01, HLA-A*32:01,  HLA-B*57:01, HLA-A*68:01, HLA-A*68:02 | 7 | -0.0184 (Probable non-antigen) | Non-allergen | Non-toxin | NEGATIVE -0.0008011012 |
| NLLPSTLDY | 1.2863 | None | 0 | 0.5976 (Probable antigen) | Allergen | Non-Toxin | NEGATIVE -0.79481912 |
| SADAAWIIF | 1.1852 | HLA-B*35:01 | 1 | 0.8244 (Probable antigen) | Non-allergen | Non-Toxin | POSITIVE 0.0055844258 |
| HSADAAWII | 1.1798 | HLA-B*58:01, HLA-A*68:02, HLA-A*02:06 | 3 | 0.8212 (Probable antigen) | Non-allergen | Non-Toxin | POSITIVE 0.081548412 |
| KLNDDTQVY | 1.0026 | HLA-B*15:01, HLA-A*30:02, HLA-A*32:01 | 3 | -0.3658 (Probable non-antigen) | Allergen | Non-Toxin | NEGATIVE -0.36284857 |
| RLKTLDIHY | 0.9206 | HLA-B*15:01, HLA-A*30:02, HLA-A*30:01 | 3 | 1.9035 (Probable antigen) | Non-allergen | Non-Toxin | POSITIVE 0.35168917 |
| SAPFDSVFY | 0.9191 | HLA-B*35:01, HLA-A*30:02 | 2 | 0.0542 (Probable non-antigen) | Allergen | Non-Toxin | NEGATIVE -0.073789323 |
| LSDLREACF | 0.9079 | None | 0 | 1.7255 (Probable antigen) | Non-allergen | Non-Toxin | NEGATIVE -0.42189004 |
| LREACFSYY | 0.8316 | None | 0 | 1.5067 (Probable antigen) | Non-allergen | Non-Toxin | NEGATIVE -0.21162008 |
| YSGEINLVH | 0.8202 | None | 0 | 0.8764 (Probable antigen) | Non-allergen | Non-Toxin | NEGATIVE -0.72581695 |
| ILFLMSQRY | 0.7904 | HLA-B*15:01, HLA-A*30:02, HLA-A*03:01, HLA-A*32:01 | 4 | 1.0469 (Probable antigen) | Non-allergen | Non-toxin | POSITIVE 0.049915841 |
